# Practical role of preoperative echocardiography in low-risk non-cardiac surgery

**DOI:** 10.3389/fcvm.2023.1088496

**Published:** 2023-01-23

**Authors:** Eun Kyoung Kim, Hong-Mi Choi, Jong-Hwan Lee, Dong Woo Han, Hye Sun Lee, Eui-Young Choi

**Affiliations:** ^1^Division of Cardiology, Department of Medicine, Heart Vascular Stroke Institute, Samsung Medical Center, Sungkyunkwan University School of Medicine, Seoul, Republic of Korea; ^2^Department of Cardiology, Cardiovascular Center, Seoul National University Bundang Hospital, Seoul National University College of Medicine, Seoul, Republic of Korea; ^3^Department of Anesthesiology and Pain Medicine, Samsung Medical Center, Sungkyunkwan University School of Medicine, Seoul, Republic of Korea; ^4^Department of Anesthesiology and Pain Medicine, Yonsei University College of Medicine, Seoul, Republic of Korea; ^5^Biostatistics Collaboration Unit, Yonsei University College of Medicine, Seoul, Republic of Korea; ^6^Division of Cardiology, Gangnam Severance Hospital, Yonsei University College of Medicine, Seoul, Republic of Korea

**Keywords:** non-cardiac surgery, cardiovascular event, echocardiography, non-cardiac surgery peri-operative, non-cardiac surgery risk stratification

## Abstract

**Background:**

Due to increased needs to reduce non-fatal as well as fatal cardiac events, preoperative echocardiography remains part of routine clinical practice in many hospitals. Data on the role of preoperative echocardiography in low-risk non-cardiac surgery (NCS) other than ambulatory surgeries do not exist. We aimed to investigate the role of preoperative echocardiography in predicting postoperative adverse cardiovascular events (CVEs) in asymptomatic patients undergoing low-risk NCS.

**Methods:**

The study population was derived from a retrospective cohort of 1,264 patients who underwent elective low-risk surgery at three tertiary hospitals from June 1, 2021, to June 30, 2021. Breast, distal bone, thyroid, and transurethral surgeries were included. Preoperative examination data including electrocardiography, chest radiography, and echocardiography were collected. The primary outcome was a composite of postoperative adverse CVEs including all-cause death, myocardial infarction, *cerebrovascular events*, newly diagnosed or acutely decompensated heart failure (HF), lethal arrhythmia such as sustained ventricular tachycardia/fibrillation, and new-onset atrial fibrillation within 30 days after the index surgery.

**Results:**

Preoperative echocardiography was performed in 503 patients (39.8%), most frequently in patients with breast surgery (73.5%), followed by transurethral (37.7%), distal bone (21.6%), and thyroid surgeries (11.9%). Abnormal findings were observed in 5.0% of patients with preoperative echocardiography. Postoperative adverse CVEs occurred in 10 (0.79%) patients. Although a history of previous HF was an independent predictor of postoperative CVE occurrence (adjusted odds ratio, aOR: 17.98; 95% confidence interval, CI: 1.21–266.71, *P* = 0.036), preoperative echocardiography did not significantly predict CVE in multivariate analysis (*P* = 0.097). However, in patients who underwent preoperative echocardiography, the presence of abnormal echocardiographic findings was independently associated with development of CVE after NCS (aOR: 23.93; 95% CI: 1.2.28–250.76, *P* = 0.008). In particular, the presence of wall motion abnormality was a strong predictor of postoperative adverse CVE.

**Conclusion:**

In real-world clinical practice, preoperative echocardiography was performed in substantial number of patients with potential cardiac risk even in low-risk NCS, and abnormal findings were independently associated with postoperative CVE. Future studies should identify patients undergoing low-risk NCS for whom preoperative echocardiography would be helpful to predict adverse CVE.

## 1. Introduction

The development of unexpected cardiovascular complications after non-cardiac surgery (NCS) is a major concern of surgeons and patients. To predict and reduce adverse cardiac events, preoperative cardiac evaluation is performed selectively according to type of surgery, individual medical condition, and functional capacity. Various predictive models have been proposed to assess the risk of cardiovascular event (CVE) after NCS (1–3). However, because the existing risk calculation models were designed to only consider the potential risk of cardiac death or myocardial infarction (MI), they are limited in predicting non-fatal CVEs, which are increasing with the greater number of surgeries performed in elderly patients with various comorbidities (4–9).

Low-risk NCS is an operation with less than 1% risk of fatal CVE and does not require preoperative cardiovascular risk assessment because there are no significant fluid shifts or physiologic stress (10). Therefore, routine preoperative echocardiography is discouraged in asymptomatic patients undergoing low-risk NCS (7, 10, 11). However, the data supporting this recommendation mainly focused on patients undergoing very low-risk surgery, such as cataract or ambulatory surgery that could be performed as day surgery (12, 13). Because data do not exist regarding the role of preoperative cardiac examination in low-risk surgeries other than ambulatory operations and there is an increased need to reduce non-fatal as well as fatal CVEs, preoperative echocardiography remains part of routine clinical practice in many hospitals. Due to the economic burden and predictive role of adverse cardiac events after surgery, the question of medical relevance remains. The aim of the study is to investigate the predictive role of preoperative echocardiography for postoperative adverse CVE in asymptomatic patients undergoing low-risk NCS that were not simple ambulatory surgeries.

## 2. Materials and methods

### 2.1. Study population and design

The study population was derived from a retrospective cohort of 1,264 patients 18–90 years of age who underwent elective low-risk NCS at one of three tertiary university hospitals from June 1, 2021, to June 30, 2021. Patients who underwent breast, thyroid, distal bone, or transurethral surgery classified as low-risk for postoperative cardiac events were included (10). Patients with current symptoms or signs requiring echocardiography, patients with a poor functional capacity of fewer than four METs, or patients with a life expectancy of less than 6 months were excluded. Based on review of medical records, a history of cardiovascular risk factors such as of hypertension, diabetes, renal dysfunction, cerebrovascular accident, dyslipidemia, and history of heart failure (HF) was obtained. A revised cardiac risk index (RCRI) score was calculated based on the patient’s medical condition (3).

### 2.2. Data collection

Data from electrocardiography (ECG), chest radiography (CXR), and transthoracic echocardiography performed as a preoperative examination were collected. Abnormal ECG was defined as the presence of dysrhythmias such as ventricular tachycardia, atrial flutter/fibrillation, or atrioventricular block more severe than second-degree Mobitz type I. Abnormal CXR was defined as the presence of pulmonary edema or significant cardiomegaly. On echocardiography, information including left ventricular (LV) dimension and ejection fraction (EF), diastolic function, wall motion abnormality, and valvular heart disease was recorded. LV systolic dysfunction was defined as an LVEF less than 50%. For assessment of LV diastolic function, mitral inflow velocity of the early phase (E) and late phase (A) during diastole, pulsed-wave Doppler-derived mitral annular velocity imaging in the septal wall (e’), left atrial volume index (LAVI), and peak tricuspid regurgitation (TR) velocity were analyzed. The presence of diastolic dysfunction was confirmed based on decreased mitral annulus velocity (septal e’ < 0.07 m/s), E/e’ > 14, enlarged LAV (LAVI > 34 mL/m^2^), and TR velocity > 2.8 m/s. If more than two parameters were positive, diastolic dysfunction was diagnosed. In cases with LVEF less than 50%, diastolic dysfunction was automatically diagnosed according to current guidelines (14). Significant valvular heart disease was stenosis or regurgitation of mitral, tricuspid, or aortic valve with a moderate degree or greater. Abnormal echocardiographic findings included LV systolic or significant diastolic dysfunction, wall motion abnormality, or significant valvular heart disease. The types of anesthesia as well as total operation and anesthesia time were also investigated.

### 2.3. Clinical outcome

The primary outcome was development of the composite of postoperative adverse CVEs including all-cause death, MI, cerebrovascular events (CVA), newly diagnosed or acutely decompensated HF, lethal arrhythmias such as sustained ventricular tachycardia/fibrillation, and new-onset atrial fibrillation within 30 days after the index surgery. MI was defined as the increase of cardiac troponin and at least one sign of coronary ischemia, new ST-T change or pathologic Q wave on ECG, new regional wall motion abnormality, or identification of coronary thrombus from angiography (15). We collected newly developed stroke/transient ischemia attack and cerebral hemorrhage as CVA event. Decompensated HF was defined based on symptoms or signs of pulmonary edema, cardiogenic shock, or abnormal systolic/diastolic dysfunction after surgery (16). The local institutional review boards of the individual hospitals approved this study, and informed consent was waived for this registry.

### 2.4. Statistical analysis

Data are presented as mean with standard deviation for continuous variables with normal distribution, as median with interquartile range for continuous variables without normal distribution, and as frequency with percentile for categorical variables. To compare the demographic, hemodynamic parameters, and endpoints between two groups, independent two-sample *t*-test was used for continuous variables and chi-square test or Fisher exact test for categorical variables as appropriate. Mann-Whitney *U* tests were performed for comparison of variables without normal distribution. Logistic regression analysis was used to determine the significant independent variables associated with development of endpoints. The significant clinical factors based on univariate analysis and clinical relevancy were included in multivariate logistic regression analysis to adjust for confounding factors. Covariates included a history of heart failure, renal dysfunction, dyslipidemia, RCRI score and abnormal chest radiography. All analyses were conducted with SPSS software version 27.0 (IBM, Chicago, IL, USA). Two-sided *P*-value < 0.05 was considered statistically significant.

## 3. Results

### 3.1. Entire cohort

**FIGURE 1:**
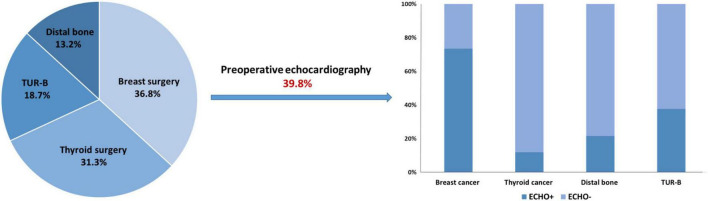
The rate of preoperative echocardiography for patients undergoing low-risk non-cardiac surgery.

A total of 1,264 consecutive patients underwent low-risk NCS consisting of 36.8% breast, 31.3% thyroid, 18.7% transurethral, and 13.2% distal bone surgeries. The mean age was 51.7 ± 14.9 years and 73.8% were female. The majority of breast and thyroid surgeries was for cancer resection (86.9% of breast surgeries and 85.6% of thyroid surgeries). Approximately 8.0% of enrolled patients had an RCRI score of 1 or 2. ECG was performed in 99.4% of patients and 10 (0.8%) had abnormal results. CXR was performed in 89.5% of patients and 94 (7.4%) showed abnormal findings. Preoperative echocardiography was performed in 503 patients (39.8%; Figure 1). The rate of preoperative echocardiography varied by type of surgery and was most frequently performed in patients with breast surgery (73.5%), followed by transurethral (37.7%), distal bone (21.6%), and thyroid surgeries (11.9%).

### 3.2. Comparison between echo- and non-echo group

Among 503 patients who had preoperative echocardiography, abnormal findings were observed in 25 (5.0%); LV systolic dysfunction in 1.8%, regional wall motion abnormality in 2.6%, significant valvular heart disease in 0.8%, and diastolic dysfunction in 2.6%. Patients who underwent preoperative echocardiography were older, more frequently female, and had a higher incidence of comorbidities such as renal dysfunction, ischemic heart disease, and previous HF than subjects without preoperative echocardiography (non-echo group, Table 1). Total operation and anesthetic times were significantly longer in patients who received preoperative echocardiography than in patients who did not (96.3 ± 79.3 *vs*. 78.7 ± 70.0 min, *P* < 0.001 for total operation time; 130.7 ± 83.3 *vs*. 109.6 ± 78.1 min, *P* < 0.001 for total anesthetic time). Patients who received preoperative echocardiography were more likely to have a higher RCRI score, indicating medically higher risk, than patients who did not receive preoperative echocardiography (12.7 *vs*. 5.0%, *P* < 0.001). In multivariate analysis, old age, female, the presence of renal dysfunction, previous ischemic heart disease, general anesthesia and longer operation time were independently associated with performing preoperative echocardiography (Table 2).

**TABLE 1:** Clinical characteristics in patients with and without preoperative echocardiography.

	No preoperative echocardiography (*N* = 761)	Preoperative echocardiography (*N* = 503)	*P*-value
Age, years	48.5 ± 14.4	56.4 ± 14.4	<0.001
Age ≥ 65 years	96 (12.6)	166 (33.0)	<0.001
Female	523 (68.7)	410 (81.5)	<0.001
BMI, kg/m^2^	24.2 ± 3.9	23.9 ± 3.4	0.127
SBP, mmHg	125 ± 17	121 ± 18	<0.001
DBP, mmHg	75 ± 12	70 ± 11	<0.001
Heart rate, beats/min	75 ± 12	72 ± 13	<0.001
Hypertension	120 (15.8)	66 (13.1)	0.193
Diabetes			0.832
OHA only	51 (6.7)	38 (7.6)	
Insulin	4 (0.5)	3 (0.6)	
Dyslipidemia	68 (8.9)	41 (8.2)	0.627
Atrial fibrillation	3 (0.4)	6 (1.2)	0.168
Renal dysfunction	14 (1.8)	35 (7.0)	<0.001
Ischemic heart disease	9 (1.2)	22 (4.4)	<0.001
Previous heart failure	0	6 (1.2)	0.004
Previous CVA	13 (1.7)	15 (3.0)	0.132
RCRI: 1 point	36 (4.7)	48 (9.5)	0.001
RCRI: 2 points	2 (0.3)	15 (3.0)	<0.001
RCRI: 3 points	0	1 (0.2)	0.398
Surgery type			<0.001
Breast	123 (16.2)	342 (68.0)	
Thyroid	349 (45.9)	47 (9.3)	
Distal bone	185 (24.3)	51 (10.1)	
Transurethral	104 (13.7)	63 (12.5)	
Total operation time, min	78.7 ± 70.0	96.3 ± 79.3	<0.001
Anesthesia			0.002
MAC	23 (3.0)	3 (0.6)	
General	655 (86.1)	460 (91.5)	
Spinal	83 (10.9)	40 (8.0)	
Total anesthesia time, min	109.6 ± 78.1	130.7 ± 83.3	<0.001

Data are presented with *n* (%) or mean ± standard deviation. BMI, body mass index; SBP, systolic blood pressure, DBP, diastolic blood pressure, CVA, cerebrovascular accident; RCRI, revised cardiac risk index; MAC, monitored anesthetic care.

**TABLE 2:** Multivariate analysis for prediction of patients performing preoperative echocardiography.

	Adjusted OR with 95% CI	*P*-value
Age > 65 years	5.72 (3.96–8.27)	<0.001
Female	3.10 (2.17–4.43)	<0.001
General anesthesia	2.29 (1.45–3.61)	<0.001
Total operation time	1.00 (1.00–1.01)	<0.001
Systolic blood pressure	0.99 (0.98–0.99)	<0.001
Heart rate	0.98 (0.97–0.99)	0.001
Renal dysfunction	6.23 (2.07–18.77)	0.001
Previous ischemic heart disease	3.98 (1.20–13.26)	0.024
Previous heart failure	NS	0.999
RCRI ≥ 1 point	0.59 (0.24–1.49)	0.267

OR, odds ration; RCRI, revised cardiac risk index.

### 3.3. Clinical outcome

Among 1,264 patients, major adverse CVE after the index surgery occurred in 10 patients (0.79%) and consisted of 1 mortality (0.08%), 6 HF (0.47%), 2 atrial fibrillation (0.16%), and 1 ventricular tachycardia (0.08%). Except for 2 patients who had newly developed atrial fibrillation, all events occurred in patients who received preoperative echocardiography. There was no CVA during follow-up. Table 3 shows the comparison of clinical characteristics between patients with and without CVEs after the index surgery. Patients who developed CVE more frequently had a history of previous HF and renal dysfunction (20.0 *vs*. 0.3%, *P* = 0.001 for previous HF and 30.0 *vs*. 3.7%, P = 0.005 for renal dysfunction). Preoperative echocardiography was more frequently performed in patients with CVE than in subjects without CVE (80.0 *vs*. 39.5%, *P* = 0.018). Among 503 patients who underwent preoperative echocardiography, abnormal echocardiographic findings were more common in patients with CVE than in subjects without CVE (62.5 *vs*. 4.0%, *P* < 0.001; Supplementary Table 1).

**TABLE 3:** Clinical characteristics based on development of adverse CVEs after NCS.

	CVE (–) (*N* = 1,254)	CVE (+) (*N* = 10)	*P*-value
Age, years	51.6 ± 14.9	61.0 ± 17.9	0.110
Female	928 (74.0)	5 (50.0)	0.139
BMI, kg/m^2^	24.0 ± 3.7	24.6 ± 1.4	0.268
SBP, mmHg	123.5 ± 17.5	125.6 ± 21.7	0.923
DBP, mmHg	72.9 ± 11.6	73.1 ± 11.8	0.894
Heart rate, beats/min	73.9 ± 12.2	80.3 ± 11.5	0.101
General anesthesia	1,106 (88.2)	9 (90.0)	1.000
Surgery type			0.061
Breast	461 (36.8)	4 (40.0)	
Thyroid	395 (31.5)	1 (10.0)	
Distal bone	235 (18.7)	1 (10.0)	
Transurethral	163 (13.0)	4 (40.0)	
Total operation time, min	85.8 ± 74.3	71.1 ± 52.3	0.507
Total anesthetic time, min	118.1 ± 81.0	103.6 ± 58.4	0.650
Previous CVA	28 (2.2)	0	1.000
Previous heart failure	4 (0.3)	2 (20.0)	0.001
Ischemic heart disease	30 (2.4)	1 (10.0)	0.221
Renal dysfunction	46 (3.7)	3 (30.0)	0.005
Atrial fibrillation	9 (0.7)	0	1.000
Dyslipidemia	106 (8.5)	3 (30.0)	0.048
Hypertension	184 (14.7)	2 (20.0)	0.648
Diabetes treated with insulin	7 (0.6)	0	1.000
RCRI ≥ 1 point	98 (7.8)	4 (40.0)	0.006
Preoperative echocardiography	495 (39.5)	8 (80.0)	0.018
Abnormal ECG findings	9 (0.7)	1 (10.0)	0.077
Abnormal CXR findings	90 (7.2)	4 (40.0)	0.004

Data are presented with *n* (%) or mean ± standard deviation. CVEs, cardiovascular events; NCS, non-cardiac surgery; BMI, body mass index; SBP, systolic blood pressure, DBP, diastolic blood pressure, CVA, cerebrovascular accident; RCRI, revised cardiac risk index; ECG, electrocardiography; CXR, chest radiography.

Although a history of previous HF was an independent predictor of postoperative CVE occurrence (adjusted odds ratio, aOR: 17.98; 95% confidence interval, CI: 1.21–266.71, *P* = 0.036), performing preoperative echocardiography did not significantly predict CVE in multivariate analysis (*P* = 0.097) among the entire population (Table 4). However, in patients who underwent preoperative echocardiography, the presence of abnormal echocardiographic findings was independently associated with development of CVE after NCS (aOR: 23.93; 95% CI: 2.28–250.76, *P* = 0.008; Table 5). In particular, wall motion abnormality was a strong predictor of postoperative adverse CVE (aOR: 14.12; 95% CI: 1.25–159.01, *P* = 0.032; Supplementary Table 2).

**TABLE 4:** Predictors of adverse CVEs after NCS in all patients.

	OR	*P*-value	Adjusted OR	*P*-value
History of heart failure	78.13 (12.48–489.08)	<0.001	17.98 (1.21–266.71)	0.036
Renal dysfunction	11.26 (2.82–44.92)	0.001	3.17 (0.18–54.97)	0.429
Dyslipidemia	4.64 (1.18–18.2)	0.028	2.67 (0.52–13.80)	0.241
RCRI ≥ 1 point	7.86 (2.18–28.34)	0.002	0.77 (0.05–12.47)	0.851
Abnormal CXR	8.62 (2.39–31.11)	0.001	4.73 (0.99–22.53)	0.051
Preoperative echocardiography	6.13 (1.30–29.0)	0.022	4.04 (0.78–21.04)	0.097

CVEs, cardiovascular events; NCS, non-cardiac surgery; OR, odds ratio; RCRI, revised cardiac risk index; CXR, chest radiography.

**TABLE 5:** Predictors of adverse CVEs after NCS in patients who underwent preoperative echocardiography.

	OR	*P*-value	Adjusted OR	*P*-value
Heart rate	1.06 (1.01–1.10)	0.019	1.07 (1.00–1.13)	0.037
Dyslipidemia	7.22 (1.66–31.36)	0.008	2.47 (0.36–17.04)	0.358
Previous heart failure	40.92 (6.25–267.77)	< 0.001	4.50 (0.24–83.33)	0.312
Renal dysfunction	8.68 (1.99–37.97)	0.004	6.13 (0.23–164.84)	0.281
RCRI ≥ 1 point	7.25 (1.77–29.75)	0.006	0.24 (0.01–9.90)	0.450
Abnormal CXR	7.43 (1.71–32.30)	0.008	0.87 (0.11–7.21)	0.898
Abnormal echocardiography	39.58 (8.84–177.34)	<0.001	23.93 (2.28–250.76)	0.008

CVEs, cardiovascular events; NCS, non-cardiac surgery; OR, odds ratio; RCRI, revised cardiac risk index; CXR, chest radiography.

### 3.4. Subgroup analysis

**FIGURE 2:**
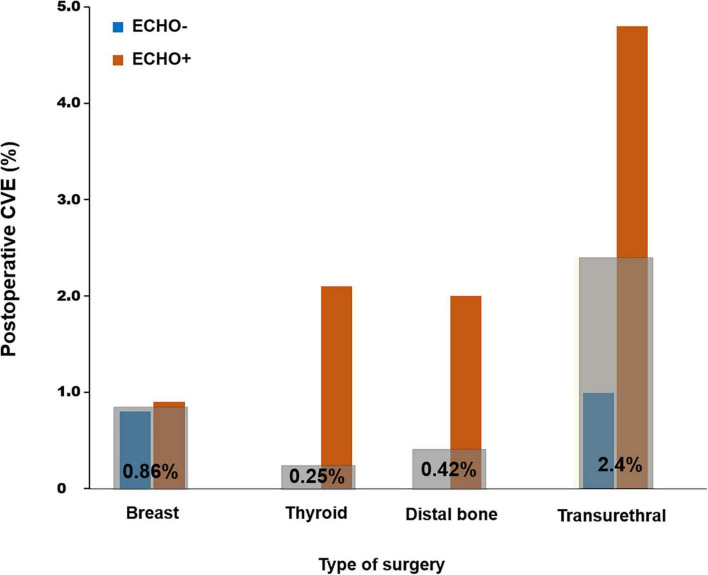
Postoperative CVE according the type of surgery. CVE, cardiovascular event.

Subgroup analysis was performed to evaluate the differences between patients who received preoperative echocardiography and those who did not based on type of surgery (Supplementary Tables 3–6). Patients who underwent breast surgery were relatively younger than subjects who underwent other types of NCS. Significant differences were not found in the previous medical histories and RCRI score between patients who did and did not receive preoperative echocardiography among those who underwent breast surgery. However, in patients who underwent thyroid, distal bone, or transurethral surgery, those who received preoperative echocardiography were significantly older and more frequently had a history of ischemic heart disease and RCRI score of 1 or higher. Postoperative CVE according to the type of surgery are presented in Figure 2.

## 4. Discussion

In this observational study, we investigated the real-world clinical practice regarding preoperative cardiac risk assessment including echocardiography in asymptomatic patients who underwent low-risk NCS. ECG and CXR were performed in most patients before surgery. Preoperative echocardiography was planned for approximately 40% of patients, and the rate of examination differed based on type of surgery. The overall incidence of adverse CVEs was as low as 0.8%. Preoperative echocardiography was more frequently performed in elderly patients and subjects who had comorbidities associated with cardiovascular disease. Therefore, adverse CVE after the surgery was more likely to occur in these patients. After adjusting for clinical factors, preoperative echocardiography did not significantly predict CVE. However, in analysis of patients who underwent preoperative echocardiography, abnormal echocardiographic findings, especially wall motion abnormality, were independent predictors of adverse CVE.

### 4.1. Actual practice in preoperative cardiac evaluation before low-risk NCS

In previous studies to confirm the clinical usefulness of preoperative echocardiography, the main focus was on mortality or MI after surgery. However, non-fatal complications such as congestive heart failure or arrhythmia are also major events that delay patient recovery and increase medical costs. Therefore, the present study intended to evaluate those fatal and non-fatal CVEs comprehensively. The incidence of adverse CVEs was less than 1%, which was consistent with data documented in the current guidelines (10, 11). However, because our data included patients from tertiary university hospitals with advanced experience in perioperative management, the rate of postoperative CVEs might have been higher if patients from secondary care hospitals were included. For low-risk NCS, the role of preoperative echocardiography in all patients or in elderly patients without comorbidities is unclear. The data showed that a history of HF was an independent predictor of CVE. In addition, abnormal echocardiographic findings were significantly associated with adverse events after surgery. Further well-designed prospective studies are needed to stratify patients undergoing low-risk NCS for whom preoperative echocardiography is beneficial.

Although our study focused on patients with non-cardiac-related symptoms and RCRI score ≤ 2 who underwent low-risk NCS, preoperative echocardiography was performed in a significant number of patients. This clinical practice is inconsistent with the current guidelines for preoperative cardiac evaluation (10, 11). There were several possible explanations for why this clinical practice was inevitable. In cases of breast surgery, which comprised the majority of patients, echocardiography might have been commonly performed due to concerns regarding LV dysfunction due to chemotherapy before and after surgery. In cases of thyroid, transurethral, or distal bone surgery, echocardiography was mainly performed in elderly patients or subjects with cardiovascular risk factors. Based on our findings, although considered low-risk NCS, preoperative echocardiography was prescribed based on patient age; underlying medical conditions that led to the operation, especially cancer; and cardiac risk factors. The results show the need for a new risk scoring system that can reflect the current clinical situation.

### 4.2. Potential benefits of preoperative echocardiography before low-risk NCS

Research on the role of preoperative echocardiography in low-risk NCS has not been previously reported. Two decades ago, Rohde L. et al. investigated the role of echocardiography for risk stratification of patients undergoing major NCS, including orthopedic and abdominal surgeries (17). The authors reported that information such as LV systolic dysfunction or significant valvular heart disease was useful for predicting major cardiac events in patients with an increased risk of cardiac complication based on clinical criteria but not in low-risk patients. However, the risk probability associated with the surgeries included in that study was heterogeneous from low-risk to high-risk, limiting conclusions in true low-risk NCS patients.

In the study data, abnormal findings were observed in 5.0% of low-risk NCS patients who underwent preoperative echocardiography, with relatively high risk and significantly associated with postoperative CVE. In contrast to previous studies (17, 18), LV systolic function and valve disease as well as LV diastolic function and wall motion abnormality were analyzed on echocardiography. Among various parameters, wall motion abnormality regardless of LV systolic dysfunction was an independent predictor of postoperative adverse CVE. Even for patients undergoing low-risk NCS, some might benefit from comprehensive preoperative echocardiography to help predict postoperative adverse CVE. Large-scale prospective data are needed to select patients at cardiac risk undergoing low-risk NCS for whom preoperative echocardiography is helpful.

### 4.3. Study limitations

The present study had several limitations. First, this was a retrospective observational study, and the results might be affected by unrecognized confounding factors. Although multivariate analysis and adjustment for several potential confounding factors were performed, unmeasured or unobserved variables, such as preoperative laboratory findings, could not be corrected. However, consecutive patients were included from multiple centers, limiting the influence of selection bias. Second, there is no validated classification of NCS for the risk of cardiac complications. Homogeneous data were analyzed by registering four types of definite low-risk surgeries commonly performed in our country. However, it is uncertain whether the study results could be generalized to all low-risk NCS. Third, because the study population was from tertiary hospitals, the predictive value of preoperative echocardiography for postoperative adverse CVEs could be different that in lower-level hospitals. Last, the number of enrolled patients was relatively small to determine statistical significance of clinical predictors for CVE. However, the data were meaningful because strict inclusion criteria were applied to accurately identify the role of preoperative echocardiography in actual low-risk NCS cases.

## 5. Conclusion

In actual clinical practice, preoperative echocardiography was performed in many patients with potential cardiac risk even in low-risk NCS. Abnormal echocardiographic findings were independently associated with postoperative CVE. Future data are needed to identify patients undergoing low-risk NCS for whom preoperative echocardiography is helpful for predicting adverse CVEs.

## Data availability statement

The original contributions presented in this study are included in the article/Supplementary material, further inquiries can be directed to the corresponding author.

## Ethics statement

The studies involving human participants were reviewed and approved by Samsung Medical Center Institutional Review Board. Written informed consent for participation was not required for this study in accordance with the national legislation and the institutional requirements.

## Author contributions

EYC, EKK, and HMC: conception and coordination of the study. EYC, EKK, HMC, HSL, and DWH: design of ethical issues. EYC, EKK, HMC, and JHL: acquisition of data. DWH, HSL, and JHL: data review. HSL: statistical analysis. All authors: manuscript preparation and manuscript approval.
