# Supplementary material for: Practical role of preoperative echocardiography in low-risk non-cardiac surgery
Source: Front Cardiovasc Med. 2023 Jan 23;10:1088496. doi: 10.3389/fcvm.2023.1088496 (PMC9899884; doi:10.3389/fcvm.2023.1088496)
Supplement: Supplementary file 1 [file Table_1.DOCX]

**Supplement Files**

**Practical role of preoperative echocardiography in low risk non-cardiac surgeries**

**Supplement Table 1.**

**Abnormal echocardiographic findings in patients who underwent preoperative echocardiography**

| **Echocardiography** | **CVE (-)** | **CVE (+)** | **P value** |
| --- | --- | --- | --- |
|  | **(N=495)** | **(N=8)** |  |
| **Abnormal echo findings** | 20 (4.0) | 5 (62.5) | <0.001 |
| **LV systolic dysfunction** | 8 (1.6) | 1 (12.5) | 0.135 |
| **Significant diastolic dysfunction** | 11 (2.2) | 2 (25.0) | 0.016 |
| **Valvular heart disease** | 3 (0.6) | 1 (12.5) | 0.062 |
| **Wall motion abnormality** | 10 (2.0) | 3 (37.5) | 0.001 |

Data are presented with n (%) or mean ± standard deviation. CVE, cardiovascular events; LV, left ventricle.

**Supplement Table 2.**

**Multivariate analysis for prediction of postoperative cardiovascular events in patients who underwent preoperative echocardiography**

|  | **Adjusted OR with 95% CI** | **P value** |
| --- | --- | --- |
| **Male** | 1.15 (0.17-7.67) | 0.888 |
| **Dyslipidemia** | 1.94 (0.27-13.92) | 0.510 |
| **RCRI ≥1 point** | 1.52 (0.18-12.93) | 0.701 |
| **Significant diastolic dysfunction** | 1.59 (0.16-16.36) | 0.696 |
| **Wall motion abnormality** | 14.12 (1.25-159.01) | 0.032 |
| **Significant valvular heart disease** | 8.819 (0.19-421.36) | 0.270 |

OR, odds ration; RCRI, Revised Cardiac Risk Index; CXR, chest radiography; LV, left ventricle.

**Supplement Table 3. Clinical characteristics in patients who underwent breast surgery**

|  | **Non-echo group**  **(N=123)** | **Echo group**  **(N=342)** | **P value** |
| --- | --- | --- | --- |
| **Age, years** | 49.9±12.0 | 50.4±11.3 | 0.944 |
| **Age≥65 years** | 16 (13.0) | 40 (11.7) | 0.701 |
| **Female** | 122 (99.2) | 342 (100) | 0.265 |
| **BMI, kg/m^2^** | 23.2±3.7 | 23.4±3.3 | 0.310 |
| **SBP, mmHg** | 122.4±17.3 | 117.4±16.7 | 0.006 |
| **DBP, mmHg** | 72.9±10.7 | 69.0±10.7 | 0.001 |
| **Heart rate, beats/min** | 77.0±11.2 | 72.5±13.0 | <0.001 |
| **Hypertension** | 21 (17.1) | 16 (4.7) | <0.001 |
| **Diabetes** | 6 (4.9) | 9 (2.6) | 0.239 |
| **Dyslipidemia** | 16 (13.0) | 7 (2.0) | <0.001 |
| **Atrial fibrillation** | - | 1 (0.3) | 1.000 |
| **Renal dysfunction** | 1 (0.8) | 5 (1.5) | 1.000 |
| **Ischemic heart disease** | - | 2 (0.6) | 1.000 |
| **Previous heart failure** | - | - |  |
| **Previous CVA** | 1 (0.8) | 3 (0.9) | 1.000 |
| **RCRI: 1 point** | 2 (1.6) | 8 (2.3) | 1.000 |
| **RCRI: 2 points** | - | 1 (0.3) | 1.000 |
| **Total operation time, min** | 146.3±112.7 | 113.2±86.4 | 0.013 |
| **General anesthesia** | 119 (96.7) | 341 (99.7) | 0.019 |
| **Total anesthetic time, min** | 183.6±125.2 | 148.0±89.7 | 0.024 |
| **Abnormal ECG** | - | 1 (0.3) | 1.000 |
| **Abnormal CXR** | 8 (6.5) | 10 (2.9) | 0.100 |
| **Abnormal echocardiography** |  | 4 (1.2%) |  |
| **LV systolic dysfunction** |  | 1 (0.3) |  |
| **LV diastolic dysfunction** |  | 3 (0.9) |  |
| **Wall motion abnormality** |  | 1 (0.3) |  |
| **Valvular heart disease** |  | - |  |
| **Postoperative CVE** | 1 (0.8) | 3 (0.9) | 1.000 |

Data are presented with n (%) or mean ± standard deviation. BMI, body mass index; SBP, systolic blood pressure, DBP, diastolic blood pressure, CVA, cerebrovascular accident; RCRI, Revised Cardiac Risk Index; ECG, electrocardiography; CXR, chest radiography; LV, left ventricle; CVE, cardiovascular events

**Supplement Table 4. Clinical characteristics in patients who underwent transurethral surgery**

|  | **Non-echo group** | **Echo group** | **P value** |
| --- | --- | --- | --- |
|  | **(N=104)** | **(N=63)** |  |
| **Age, years** | 63.8±12.3 | 74.9±7.4 | <0.001 |
| **Age≥65 years** | 45 (43.3) | 61 (96.8) | <0.001 |
| **Female** | 21 (20.2) | 8 (12.7) | 0.215 |
| **BMI, kg/m^2^** | 24.3±3.5 | 24.0±2.6 | 0.582 |
| **SBP, mmHg** | 130.3±17.8 | 123.7±16.1 | 0.018 |
| **DBP, mmHg** | 76.5±11.7 | 70.0±10.5 | <0.001 |
| **Heart rate, beats/min** | 75.3±12.4 | 69.0±11.0 | 0.001 |
| **Hypertension** | 25 (24.0) | 15 (23.8) | 0.973 |
| **Diabetes** | 16 (15.4) | 14 (22.2) | 0.309 |
| **Dyslipidemia** | 7 (6.7) | 12 (19.0) | 0.015 |
| **Atrial fibrillation** | 3 (2.9) | 2 (3.2) | 1.000 |
| **Renal dysfunction** | 4 (3.8) | 20 (31.7) | <0.001 |
| **Ischemic heart disease** | 6 (5.8) | 10 (15.9) | 0.032 |
| **Previous heart failure** | - | 4 (6.3) | 0.019 |
| **Previous CVA** | 5 (4.8) | 7 (11.1) | 0.137 |
| **RCRI: 1 point** | 17 (16.3) | 24 (38.1) | 0.002 |
| **RCRI: 2 points** | - | 7 (11.1) | 0.001 |
| **Total operation time, min** | 40.0±35.6 | 25.7±17.7 | 0.017 |
| **General anesthesia** | 82 (78.8) | 54 (85.7) | 0.269 |
| **Total anesthetic time, min** | 63.1±37.2 | 52.7±21.0 | 0.239 |
| **Abnormal ECG** | 4 (3.8) | 2 (3.2) | 1.000 |
| **Abnormal CXR** | 12 (11.5) | 11 (17.5) | 0.282 |
| **Abnormal echocardiography** |  | 10 (15.9) |  |
| **LV systolic dysfunction** |  | 3 (4.8) |  |
| **LV diastolic dysfunction** |  | 2 (3.2) |  |
| **Wall motion abnormality** |  | 7 (11.1) |  |
| **Valvular heart disease** |  | 2 (3.2) |  |
| **Postoperative CVE** | 1 (1.0) | 3 (4.8) | 0.151 |

Data are presented with n (%) or mean ± standard deviation. BMI, body mass index; SBP, systolic blood pressure, DBP, diastolic blood pressure, CVA, cerebrovascular accident; RCRI, Revised Cardiac Risk Index; ECG, electrocardiography; CXR, chest radiography; LV, left ventricle; CVE, cardiovascular events

**Supplement Table 5. Clinical characteristics in patients who underwent thyroid surgery**

|  | **Non-echo group** | **Echo group** | **P value** |
| --- | --- | --- | --- |
|  | **(N=349)** | **(N=47)** |  |
| **Age, years** | 43.8±11.4 | 60.3±11.8 | 0.001 |
| **Age≥65 years** | 15 (4.3) | 22 (46.8) | <0.001 |
| **Female** | 281 (80.5) | 26 (55.3) | <0.001 |
| **BMI, kg/m^2^** | 24.0±4.1 | 25.7±4.0 | 0.001 |
| **SBP, mmHg** | 122.4±15.4 | 127.7±17.1 | 0.049 |
| **DBP, mmHg** | 74.6±11.5 | 73.9±10.9 | 0.796 |
| **Heart rate, beats/min** | 75.0±10.9 | 71.3±12.1 | 0.004 |
| **Hypertension** | 38 (10.9) | 15 (3.8) | <0.001 |
| **Diabetes** | 13 (3.7) | 4 (8.5) | 0.007 |
| **Dyslipidemia** | 32 (9.2) | 10 (2.5) | 0.020 |
| **Atrial fibrillation** | - | 1 (2.1) | 0.119 |
| **Renal dysfunction** | 4 (1.1) | 1 (2.1) | 0.470 |
| **Ischemic heart disease** | 1 (0.3) | 5 (10.6) | <0.001 |
| **Previous heart failure** | - | 1 (2.1) | 0.119 |
| **Previous CVA** | 1 (0.3) | 1 (2.1) | 0.224 |
| **RCRI: 1 point** | 6 (1.7) | 7 (14.9) | <0.001 |
| **RCRI: 2 points** | - | 1 (2.1) | 0.119 |
| **Total operation time, min** | 72.0±47.3 | 87.9±48.4 | 0.008 |
| **General anesthesia** | 349 (100) | 47 (100) | - |
| **Total anesthetic time, min** | 99.0±52.6 | 120.3±55.8 | 0.002 |
| **Abnormal ECG** | - | 1 (2.1) | 0.119 |
| **Abnormal CXR** | 14 (4.0) | 8 (17.0) | 0.002 |
| **Abnormal echocardiography** |  | 2 (4.3) |  |
| **LV systolic dysfunction** |  | 2 (4.3) |  |
| **LV diastolic dysfunction** |  | 1 (2.1) |  |
| **Wall motion abnormality** |  | 2 (4.3) |  |
| **Valvular heart disease** |  | - |  |
| **Postoperative CVE** | - | 1(2.1) | 0.119 |

Data are presented with n (%) or mean ± standard deviation. BMI, body mass index; SBP, systolic blood pressure, DBP, diastolic blood pressure, CVA, cerebrovascular accident; RCRI, Revised Cardiac Risk Index; ECG, electrocardiography; CXR, chest radiography; LV, left ventricle; CVE, cardiovascular events

**Supplement Table 6. Clinical characteristics in patients who underwent distal bone surgery**

|  | **Non-echo group** | **Echo group** | **P value** |
| --- | --- | --- | --- |
|  | **(N=185)** | **(N=51)** |  |
| **Age, years** | 48.1±15.7 | 70.6±11.1 | <0.001 |
| **Age≥65 years** | 20 (10.8) | 43 (84.3) | <0.001 |
| **Female** | 99 (53.5) | 34 (66.7) | 0.094 |
| **BMI, kg/m^2^** | 25.0±3.7 | 25.1±2.6 | 0.604 |
| **SBP, mmHg** | 128.6±17.5 | 137.8±19.6 | 0.002 |
| **DBP, mmHg** | 75.3±12.6 | 73.9±10.1 | 0.332 |
| **Heart rate, beats/min** | 74.4±12.8 | 71.7±13.0 | 0.171 |
| **Hypertension** | 36 (19.5) | 20 (39.2) | 0.003 |
| **Diabetes** | 18 (9.7) | 13 (25.5) | 0.012 |
| **Dyslipidemia** | 13 (7.0) | 12 (23.5) | 0.001 |
| **Atrial fibrillation** | - | 2 (3.9) | 0.046 |
| **Renal dysfunction** | 5 (2.7) | 9 (17.6) | <0.001 |
| **Ischemic heart disease** | 2 (1.1) | 5 (9.8) | 0.006 |
| **Previous heart failure** | - | 1 (2.0) | 0.216 |
| **Previous CVA** | 6 (3.2) | 4 (7.8) | 0.229 |
| **RCRI: 1 point** | 11 (5.9) | 9 (17.6) | 0.019 |
| **RCRI: 2 points** | 2 (1.1) | 6 (11.8) | 0.002 |
| **Total operation time, min** | 68.6±51.0 | 78.5±33.1 | 0.008 |
| **General anesthesia** | 105 (56.8) | 18 (35.3) | 0.010 |
| **Total anesthetic time, min** | 106.7±60.5 | 120.0±38.7 | 0.005 |
| **Abnormal ECG** | - | 2 (3.9) | 0.046 |
| **Abnormal CXR** | 20 (10.8) | 11 (21.6) | 0.044 |
| **Abnormal echocardiography** |  | 9 (17.6) |  |
| **LV systolic dysfunction** |  | 3 (5.9) |  |
| **LV diastolic dysfunction** |  | 7 (13.7) |  |
| **Wall motion abnormality** |  | 3 (5.9) |  |
| **Valvular heart disease** |  | 2 (3.9) |  |
| **Postoperative CVE** | - | 1(2.0) | 0.216 |

Data are presented with n (%) or mean ± standard deviation. BMI, body mass index; SBP, systolic blood pressure, DBP, diastolic blood pressure, CVA, cerebrovascular accident; RCRI, Revised Cardiac Risk Index; ECG, electrocardiography; CXR, chest radiography; LV, left ventricle; CVE, cardiovascular events
